# Time- and radiation-dose dependent changes in the plasma proteome after total body irradiation of non-human primates: Implications for biomarker selection

**DOI:** 10.1371/journal.pone.0174771

**Published:** 2017-03-28

**Authors:** Stephanie D. Byrum, Marie S. Burdine, Lisa Orr, Samuel G. Mackintosh, Simon Authier, Mylene Pouliot, Martin Hauer-Jensen, Alan J. Tackett

**Affiliations:** 1 Department of Biochemistry and Molecular Biology, University of Arkansas for Medical Sciences, Little Rock, Arkansas, United States of America; 2 CIToxLAB, Laval, Quebec, Canada; 3 Division of Radiation Health, University of Arkansas for Medical Sciences, Little Rock, Arkansas, United States of America; Northwestern University Feinberg School of Medicine, UNITED STATES

## Abstract

Acute radiation syndrome (ARS) is a complex multi-organ disease resulting from total body exposure to high doses of radiation. Individuals can be exposed to total body irradiation (TBI) in a number of ways, including terrorist radiological weapons or nuclear accidents. In order to determine whether an individual has been exposed to high doses of radiation and needs countermeasure treatment, robust biomarkers are needed to estimate radiation exposure from biospecimens such as blood or urine. In order to identity such candidate biomarkers of radiation exposure, high-resolution proteomics was used to analyze plasma from non-human primates following whole body irradiation (Co-60 at 6.7 Gy and 7.4 Gy) with a twelve day observation period. A total of 663 proteins were evaluated from the plasma proteome analysis. A panel of plasma proteins with characteristic time- and dose-dependent changes was identified. In addition to the plasma proteomics study reported here, we recently identified candidate biomarkers using urine from these same non-human primates. From the proteomic analysis of both plasma and urine, we identified ten overlapping proteins that significantly differentiate both time and dose variables. These shared plasma and urine proteins represent optimal candidate biomarkers of radiation exposure.

## Introduction

In today’s society, there is a distinct threat of terrorist attacks from chemical, biological, radiological, and/or nuclear weapons. Populations that are exposed to high radiation doses develop acute radiation syndrome (ARS), an acute illness caused by whole body exposure to a high dose of penetrating radiation over a short interval. Exposure to high ionizing radiation levels is associated with a wide range of biological effects including damage to macromolecules (e.g., DNA, proteins and lipids), increased mitochondria-dependent generation of reactive oxygen species (ROS), apoptosis, stress-related responses, and defects in the ability of cells to divide properly leading to an onset of symptoms. The range and severity of symptoms are dose dependent where lower doses tend to affect the hematopoietic system and gastrointestinal tract, while higher doses can result in cardiovascular and neurological effects and rapid death. Cell death from radiation is directly proportional to the mitotic rate and indirectly proportional to the cell’s state of differentiation. Areas of the body most affected by total body irradiation include the hematopoietic system, intestines, skin, spermatogenic cells and vascular system.

To effectively use existing therapies and even design new drugs as radiation countermeasures, suitable biomarkers that can be used to quantify radiation exposure (time and dose) are essential. Readily accessible biological samples for biomarker detection include urine and plasma [1]. A recent study compared the ability of plasma and urine to serve as sources of biomarkers for acute kidney injury (AKI) and concluded that the biomarkers identified in plasma had better discriminative power for preoperative risk stratification and early postoperative detection of AKI [2]. Our group previously reported a set of significantly differentiating urine proteins identified by high-resolution mass spectrometry from a study of twenty-four *Rhesus macaque* animals exposed to two different levels of gamma-irradiation (Co-60 at 6.7 Gy and 7.4 Gy) with a twelve day observation period [3]. In the current study, we examined the plasma from the same animals and identified a series of complementary biomarkers of radiation exposure. The overlapping plasma and urine candidate biomarkers are the first identified panel of putative biomarkers of radiation exposure in the *Rhesus macaque* model, which has immediate implications for human health and potential application in biodosimetry.

## Materials and methods

### Animals

The animals used in this study have been previously reported by Byrum *et al*. 2016 [3]. Briefly, the *Rhesus* monkey was selected for the study due to the fact it is a well characterized model for ARS and is the most commonly studied animal in radiation research under the US FDA Animal Rule. Procedures involving the care and use of animals in this study were reviewed and approved by the Institutional Animal Care and Use Committee (IACUC) prior to conduct. During this study the care and use of animals were conducted in accordance with the principles outlined in the current Guidelines. The contract research organization (CRO) conducting the study was and is accredited by AAALAC.

There were 24 animals included in the study, which consisted of 4 animals per radiation dose (0, 6.7, and 7.4 Gy) and day of necropsy (day 4 and day 7). There were only 2 animals per radiation dose (6.7 and 7.4 Gy) for necropsy day 12. In accordance to historical data from irradiated and control animals, we selected radiation levels of 6.7 Gy and 7.4 Gy to cause significant GI-injury while insuring survival of some animals out to 12 days post-irradiation. The days 4 and 7 represent the time points for major GI-injury, while day 12 is a later endpoint to investigate GI-injury repair. Whole-body irradiation was performed as previously described. In brief, the animals were exposed to a single uniform total body dose of gamma radiation from a Co—60 source (Theratron1000) at a dose rate of approximately 60 cGy/min for 12 minutes.

Animal numbers were selected based on the planned statistical analyses, assuming that all responses analyzed will be approximately normally distributed. With 4 animals per dose group on necropsy days 4 and 7, and 2 animals per dose group on necropsy day 12, there will be approximately 80% power to detect dose and time effects with a significance level of 5%, assuming differences of 1 standard deviation in responses between adjacent doses and between adjacent necropsy days.

All animals were monitored throughout the study period. Clinical observations were recorded on all animals twice daily until Day 3 and 4 times daily from Day 4 to 12 (at least every 6 hours). Clinical observations included particular attention to infections, hemorrhage, mucositis, diarrhea, emesis, and individual food intake. A detailed clinical examination was performed on each animal prior to animal assignment, prior to irradiation and every 3 days thereafter, including the day of irradiation (prior to irradiation) and prior to necropsy.

Room environments were controlled and continuously monitored to maintain air temperature, relative humidity, consistent light dark cycles, and 10–15 air changes per hour. All animals were housed individually in stainless steel monkey cages equipped with an automatic watering system. Municipal tap water (which was exposed to ultraviolet light and purified by reverse osmosis) were provided to the animals *ad libitum*, except during designated procedures, via an automatic watering system or water bottles. A standard certified commercial chow (Harlan Teklad Certified Hi-Fiber Primate Diet #7195C) was provided to the animals twice daily and removed overnight prior to irradiation. Treats or fresh fruits/vegetables were provided as part of the animal enrichment program.

To minimize distress, all animals were given an acclimation period of at least 1 week following their arrival to the facility and prior to the start of irradiation. Animals were also acclimated the radiotherapy chair and transportation process. Positive reinforcement consisting of non-human primate treats and/or fruit juice were utilized to facilitate acclimation. The Clinical Veterinarian monitored all animal health assessments. In the event an animal became ill, supportive care would be offered for the following indications: 1.) pain, 2.) weight loss >15% of body weight, 3.) dehydration, 4.) fever, and/or 5.) bacterial infection.

Protocols were in place that allowed for the early euthanasia of animals in unrelievable pain or distress. Clinical criteria for premature euthanasia were: 1.) respiratory distress, 2.) anorexia (for at least 3 days), 3.) weight loss in excess of 20% of body weight, 4.) unresponsiveness, 5.) acute blood loss (>10% overall estimated blood volume), 6.) seizure activity, and 7.) abnormal appearance. Animals were to be euthanized if one of the above criteria were met. Euthanasia procedures were the same for animals prematurely euthanized or euthanized at specified study endpoints. For euthanasia, the animals were sedated with an intramuscular injection of a combination of ketamine hydrochloride and acepromazine, and then euthanized by an intravenous overdose of sodium pentobarbital, followed by exsanguination. There were no illnesses, premature deaths, or early euthanasia of any animals in the study.

### Plasma collection

The 24 *Rhesus macaque* plasma samples included in this study correspond directly with the previously published urine samples [3]. Venous samples were observed from a peripheral vein (i.e. femoral or cephalic veins). Each blood sample was collected into tubes containing K2 EDTA as anticoagulant and kept on wet ice pending centrifugation. Within 30 minutes of collection, blood samples were centrifuged under refrigeration (set to +4°C at 1500 g RCF) for targeted 10 minutes. Plasma was transferred into 4 separate tubes, placed on dry ice pending storage in a freezer set to -70°C.

### High-resolution proteomics

**Fig 1:**
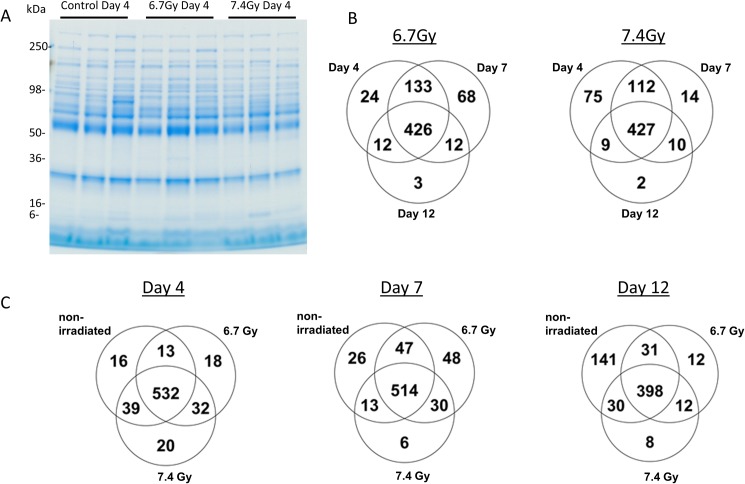
The plasma proteome from non-human primates following gamma-irradiation was analyzed by high-resolution mass spectrometry. A) Representative gel image of replicate samples from the day 4 time point under varying gamma-irradiation doses of 0 Gy, 6.7 Gy, and 7.4 Gy. Plasma was first depleted of the top 20 most abundant plasma proteins, resolved by SDS-PAGE and visualized by Coomassie-staining. B) The Venn diagrams show the number of shared and unique proteins at days 4, 7, and 12 post-irradiation at both 6.7 Gy and 7.4 Gy radiation exposures. C) The number of shared and unique proteins identified in a dose-dependent analysis (non-irradiated, 6.7 Gy, and 7.4 Gy exposures) at each time point (days 4, 7, 12).

The top 20 most abundant proteins (albumin, IgG, transferrin, fibrinogen, IgA, α2-Marcroglobulin, IgM, α1-Antitrypsin, complement C3, haptoglobulin, apolipoprotein A1, A3 and B; α1-Acid Glycoprotein, ceruloplasmin, complement C4, C1q; IgD, prealbumin, and plasminogen) in the plasma were removed using the ProteoPrep 20 Plasma Immunodepletion Kit (Sigma-Aldrich, PROT20). Twenty micrograms of protein from depleted plasma was resolved by 4–20% Tris-Glycine SDS-PAGE (Life Technologies) and the proteins were visualized by Coomassie-staining (Fig 1A). Each gel lane was sliced into 20 bands 2 mm in size. Gel bands were destained (50% methanol, 100 mM ammonium bicarbonate), followed by treatment with 10 mM Tris[2-carboxyethyl] phosphine to reduce protein disulfide bonds and 50 mM iodoacetamide to block reformation of cysteine disulfide bonds. Gel slices were then dehydrated in acetonitrile, followed by addition of 100 ng porcine sequencing grade modified trypsin in 100 mM ammonium bicarbonate and incubated at 37°C for 12–16 hours. Peptide products were then acidified in 0.1% formic acid. Tryptic peptides were separated by reverse phase Jupiter Proteo resin (Phenomenex) on a 100 × 0.075 mm column using a nanoAcquity UPLC system (Waters). Peptides were eluted using a 40 min gradient from 97:3 to 35:65 buffer A:B ratio. [Buffer A = 0.1% formic acid, 0.5% acetonitrile; buffer B = 0.1% formic acid, 75% acetonitrile]. Eluted peptides were ionized by electrospray (1.9 kV) followed by MS/MS analysis using collision induced dissociation on an LTQ Orbitrap Velos mass spectrometer (Thermo Scientific). MS data were acquired using the FTMS analyzer in profile mode at a resolution of 60,000 over a range of 375 to 1500 m/z. MS/MS data were acquired for the top 15 peaks from each MS scan using the ion trap analyzer in centroid mode and normal mass range with normalized collision energy of 35.0.

### Data analysis

Proteins were identified as reported in Byrum *et al*. 2016 [3]. The mass spectrometric raw data was analyzed by MaxQuant using a label-free intensity based approach. Proteins were identified by searching against the UniProtKB database (2015_06 release; restricted to *Rhesus macaque*; 69,970 entries). The MS1 intensities were normalized by using the intensity-based absolute quantification (iBAQ) method implemented in MaxQuant [4, 5]. Quantitative testing was performed in Scaffold Q+S using iBAQ normalized intensity values to detect differential presence of a protein between different samples (time-dependent: days 4, 7, and 12 for both radiation doses 6.7Gy and 7.4Gy; dose-dependent: non-irradiated, 6.7Gy, and 7.4Gy from days 4, 7, and 12). Proteins were tested for significance by Kruskal-Wallis with Benjamini-Hochberg correction in a time and dose dependent manner. Proteins in the time dependent analysis were considered to be significant at p < 0.00509 and p < 0.00653 (corrected Benjamini-Hochberg p-value) for dose levels 6.7 Gy and 7.4 Gy gamma-irradiation exposure. Proteins in the dose-dependent analysis were considered to be significant at p < 0.00179, p < 0.00622, and p < 0.00525 (corrected Benjamini-Hochberg p-value) for days 4, 7, and 12 post-irradiation. The log2 normalized intensity values for each data comparison were visualized by Hierarchical Clustering Explorer (HCE 3.0). The hierarchical clusters were constructed using the average linkage method with Euclidean distance measure in order to cluster the significant proteins and visually inspect trends in the data. Significant proteins were analyzed for pathway expression using QIAGEN’s Ingenuity Pathway Analysis (IPA, QIAGEN Redwood City, www.qiagen.com/ingenuity). Scaffold files from urine (data from [3]) and plasma proteomes were used to identify the total number of proteins found in both body fluids as well as proteins significantly differentiating all sample groups by the Analysis of Variance with normalization by iBAQ and multiple test correction by Benjamini-Hochberg using Scaffold’s Quantitative Analysis tool. Raw data used for all calculations can be found in S1 and S2 Tables.

## Results

We investigated changes in plasma proteome in response to total body gamma-irradiation in 24 *Rhesus macaque*. The plasma was collected and the top 20 most abundant plasma proteins were depleted prior to SDS-PAGE and mass spectrometric analysis (Fig 1A). Using MaxQuant and Scaffold programs, we identified a total of 663 proteins (338,187 spectra) from all 24 samples with a 95% protein probability, a minimum number of peptides of 2, and a 50% peptide probability threshold. The total number of identified proteins from each sample group is displayed in Venn diagrams for both time-dependent and dose-dependent analyses (Fig 1B & 1C). In the time-dependent analysis the number of proteins uniquely identified in the plasma samples was greatest at day 7 (68 proteins) with 6.7 Gy radiation exposure as compared with the higher dose of radiation in which the majority of uniquely identified proteins were identified on day 4 (75 proteins) (Fig 1B). In addition the dose-dependent analysis also shows the majority of uniquely identified proteins are found at day 7 (48 proteins) for the 6.7 Gy exposure but are identified at day 4 (20 proteins) for the 7.4 Gy gamma-irradiation exposure. This indicates more unique proteins are released in the blood due to gamma-irradiation damage at an earlier time point with the higher radiation dose level. These data suggest that certain windows of time have a greater probability for detection of plasma biomarkers of radiation exposure, and that with increasing dose these windows of time are closer to the time of exposure.

**Fig 2:**
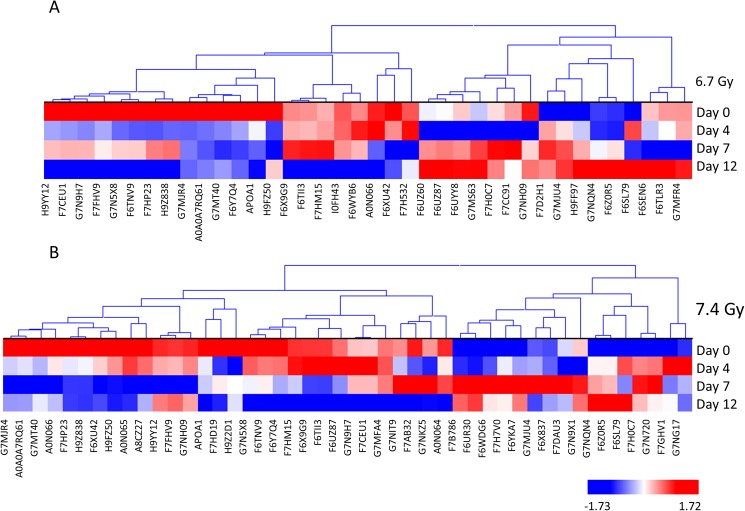
**Hierarchical cluster of significantly differentiating proteins in a time dependent manner at 6.7 Gy (A) and 7.4 Gy (B) gamma-irradiation exposures.** Proteins were clustered using the average linkage method and Euclidean distance metric. Proteins were considered significant by Kruskal-Wallis p-value corrected using Benjamini-Hochberg. Red indicates elevated levels, while blue indicates lower levels of the given protein.

**Fig 3:**
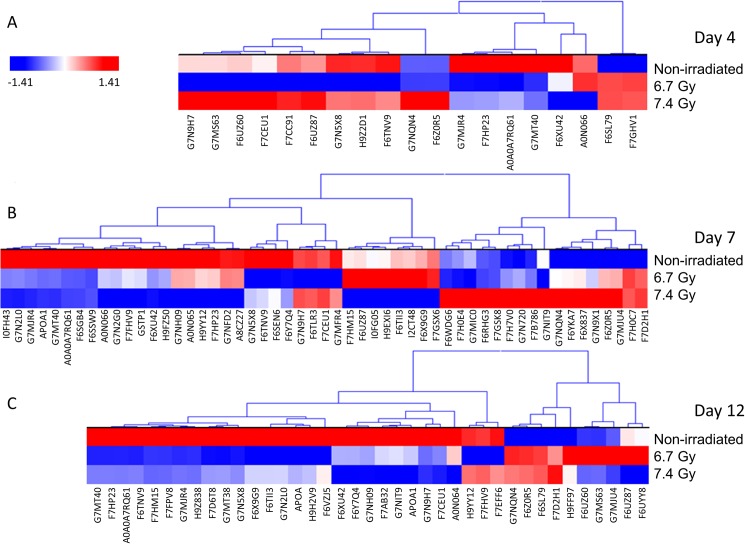
**Hierarchical cluster of significantly differentiating proteins in a dose dependent manner from days 4 (A), 7 (B), and 12 (C) post-irradiation.** Proteins were clustered using the average linkage method and Euclidean distance metric. Proteins were considered significant by Kruskal-Wallis p-value corrected using Benjamini-Hochberg. Red indicates elevated levels, while blue indicates lower levels of the given protein.

A label-free mass spectrometric workflow was used to measure relative levels of the proteins identified among the samples. The non-irradiated samples collected on day 4 served as a baseline control and were compared with both 6.7 Gy and 7.4 Gy gamma-irradiated animals for three post-irradiation time points (days 4, 7, and 12). The proteins were identified using MaxQuant and normalized by iBAQ. The log_2_ normalized intensity values were then analyzed using Kruskal-Wallis with Benjamini-Hochberg multiple test correction for each comparison. Proteins that were found to be significant after p-value correction are listed in Tables 1 and 2. The iBAQ normalized intensity values for all proteins and all samples are listed in S1 Table. Hierarchical cluster heat maps were generated using the log_2_ normalized intensity values to visually inspect trends in the data from the significantly differentiating proteins in a time-dependent and dose-dependent analysis (Figs 2 and 3). The heat maps show clear differences in protein levels from each comparison. Fig 2 shows the significant proteins from the time-dependent analysis and reveals a trend of proteins changing levels from day 4 to day 12 post-irradiation. For both radiation doses, several protein level changes are observed by day 4. In the dose-dependent analysis, the 6.7 and 7.4 Gy exposures show proteins changing in levels starting on day 4 and continuing through day 12 (Fig 3).

**Table 1:** Significantly differentiating proteins from days 4, 7, and 12 at both gamma-irradiation doses of 6.7 Gy and 7.4 Gy.

6.7 Gy			7.4 Gy		
Identified Proteins	Accession Number	p-value	Identified Proteins	Accession Number	p-value
Alpha-1-antichymotrypsin	F6SL79	0.0001	Alpha-2-macroglobulin	G7N5X8	0.0001
Alpha-1-antitrypsin	F6SEN6	0.0001	Amine oxidase	G7NIT9	0.0001
Alpha-2-macroglobulin	G7N5X8	0.0001	Apolipoprotein A-I	APOA1	0.0001
Annexin	F7H0C7	0.0001	Fibronectin isoform 4 preproprotein	H9YY12	0.0001
Antithrombin III	A0N066	0.0001	Putative uncharacterized protein	G7N9H7	0.0001
Apolipoprotein A-I	APOA1	0.0001	Putative uncharacterized protein	G7MJR4	0.0001
Fibrinogen alpha chain	F6UZ60	0.0001	Putative uncharacterized protein	G7NQN4	0.0001
Fibronectin isoform 4 preproprotein	H9YY12	0.0001	Putative uncharacterized protein	G7MT40	0.0001
Plasminogen	F7CC91	0.0001	Transferrin	A0A0A7RQ61	0.0001
Putative uncharacterized protein	G7N9H7	0.0001	Uncharacterized protein (Fragment)	F6XU42	0.0001
Putative uncharacterized protein	G7MJR4	0.0001	Uncharacterized protein	F6TNV9	0.0001
Putative uncharacterized protein	G7NQN4	0.0001	Uncharacterized protein	F7CEU1	0.0001
Putative uncharacterized protein	G7MT40	0.0001	Uncharacterized protein	F6WDG6	0.0001
Putative uncharacterized protein	G7MS63	0.0001	Uncharacterized protein	F6UZ87	0.0001
Transferrin	A0A0A7RQ61	0.0001	Uncharacterized protein	F7FHV9	0.0001
Uncharacterized protein (Fragment)	F6XU42	0.0001	Uncharacterized protein	F6Z0R5	0.0001
Uncharacterized protein	F6TNV9	0.0001	Uncharacterized protein	F6Y7Q4	0.0001
Uncharacterized protein	F7HM15	0.0001	Uncharacterized protein	F6X9G9	0.0001
Uncharacterized protein	F7CEU1	0.0001	Uncharacterized protein	F6TII3	0.0001
Uncharacterized protein	F6UZ87	0.0001	Putative uncharacterized protein	G7MJU4	0.0002
Uncharacterized protein	F6UYY8	0.0001	Alpha-1-antichymotrypsin	F6SL79	0.00021
Uncharacterized protein	F7FHV9	0.0001	Coagulation factor X protein	A0N065	0.00021
Uncharacterized protein	F6Z0R5	0.0001	Uncharacterized protein	F6X837	0.00022
Uncharacterized protein	F6Y7Q4	0.0001	Uncharacterized protein	F7DAU3	0.00023
Uncharacterized protein	F6X9G9	0.0001	Uncharacterized protein	F7GHV1	0.00024
Uncharacterized protein	F6TII3	0.0001	Uncharacterized protein	F7HM15	0.00035
Vinculin isoform VCL	I0FH43	0.0001	Uncharacterized protein	F7AB32	0.00035
Uncharacterized protein	F7HP23	0.00024	C3 and PZP-like alpha-2-macroglobulin domain-containing protein 4	G7NG17	0.00038
Complement C1r subcomponent	H9FZ50	0.00046	Prothrombin	A0N064	0.0007
Gelsolin isoform a	H9Z838	0.0011	Coagulation factor IX protein	A8CZ27	0.0013
Putative uncharacterized protein	G7NH09	0.0013	Annexin	F7H0C7	0.0014
Annexin	F7D2H1	0.0019	Putative uncharacterized protein	G7NH09	0.0015
Uncharacterized protein	F6WYB6	0.0022	Cytokeratin-1	F7B786	0.0017
Uncharacterized protein	F7H532	0.0023	Collagen alpha-2(I) chain	H9Z2D1	0.002
Angiotensinogen preproprotein	G7MFR4	0.0027	Uncharacterized protein	F7HP23	0.0021
Putative: complement C4-A isoform 1 (Fragment)	H9FF97	0.0028	Intercellular adhesion molecule 1	G7NKZ5	0.0022
Putative uncharacterized protein	G7MJU4	0.0031	Putative uncharacterized protein	G7MFA4	0.0025
Uncharacterized protein	F6TLR3	0.0045	Putative uncharacterized protein	G7N9X1	0.0032
			Uncharacterized protein	F6UR30	0.0034
			Desmoplakin-3	F7H7V0	0.0036
			Gelsolin isoform a	H9Z838	0.004
			Uncharacterized protein	F6YKA7	0.0045
			Antithrombin III	A0N066	0.0047
			Complement C1r subcomponent	H9FZ50	0.005
			Cytokeratin-6C	G7N720	0.0054
			Uncharacterized protein (Fragment)	F7HD19	0.0058

Proteins were considered significant with p-values less than the Kruskal-Wallis Benjamini-Hochberg corrected values (p < 0.00509 and p < 0.00653 for dose levels 6.7 Gy and 7.4 Gy).

**Table 2:** Significantly differentiating proteins by gamma-irradiation dose levels of 6.7 Gy and 7.4 Gy at days 4, 7, and 12.

Day 4			Day 7			Day 12		
Identified Proteins	Accession Number	p-value	Identified Proteins	Accession Number	p-value	Identified Proteins	Accession Number	p-value
Alpha-2-macroglobulin	G7N5X8	0.0001	Alpha-2-macroglobulin	G7N5X8	0.0001	Alpha-2-macroglobulin	G7N5X8	0.0001
Fibrinogen alpha chain	F6UZ60	0.0001	Amine oxidase	G7NIT9	0.0001	Apolipoprotein A-I	APOA1	0.0001
Plasminogen	F7CC91	0.0001	Annexin	F7H0C7	0.0001	Fibrinogen alpha chain	F6UZ60	0.0001
Putative uncharacterized protein	G7N9H7	0.0001	Apolipoprotein A-I	APOA1	0.0001	Fibronectin isoform 4 preproprotein	H9YY12	0.0001
Putative uncharacterized protein	G7MJR4	0.0001	Desmoplakin-3	F7H7V0	0.0001	Putative uncharacterized protein	G7N9H7	0.0001
Putative uncharacterized protein	G7NQN4	0.0001	Putative uncharacterized protein	G7N9H7	0.0001	Putative uncharacterized protein	G7MJR4	0.0001
Putative uncharacterized protein	G7MT40	0.0001	Putative uncharacterized protein	G7MJR4	0.0001	Putative uncharacterized protein	G7NQN4	0.0001
Putative uncharacterized protein	G7MS63	0.0001	Putative uncharacterized protein	G7NQN4	0.0001	Putative uncharacterized protein	G7MT40	0.0001
Transferrin	A0A0A7RQ61	0.0001	Putative uncharacterized protein	G7MT40	0.0001	Putative uncharacterized protein	G7MS63	0.0001
Uncharacterized protein (Fragment)	F6XU42	0.0001	Transferrin	A0A0A7RQ61	0.0001	Transferrin	A0A0A7RQ61	0.0001
Uncharacterized protein	F6TNV9	0.0001	Uncharacterized protein (Fragment)	F6XU42	0.0001	Uncharacterized protein (Fragment)	F6XU42	0.0001
Uncharacterized protein	F7CEU1	0.0001	Uncharacterized protein	F6TNV9	0.0001	Uncharacterized protein	F6TNV9	0.0001
Uncharacterized protein	F6UZ87	0.0001	Uncharacterized protein	F7HM15	0.0001	Uncharacterized protein	F7HM15	0.0001
Uncharacterized protein	F6Z0R5	0.0001	Uncharacterized protein	F7CEU1	0.0001	Uncharacterized protein	F7CEU1	0.0001
Alpha-1-antichymotrypsin	F6SL79	0.00017	Uncharacterized protein	F6WDG6	0.0001	Uncharacterized protein	F6UZ87	0.0001
Collagen alpha-2(I) chain	H9Z2D1	0.0008	Uncharacterized protein	F6Z0R5	0.0001	Uncharacterized protein	F6UYY8	0.0001
Uncharacterized protein	F7GHV1	0.00099	Uncharacterized protein	F6Y7Q4	0.0001	Uncharacterized protein	F7FHV9	0.0001
Uncharacterized protein	F7HP23	0.0011	Vinculin isoform VCL	I0FH43	0.0001	Uncharacterized protein	F6Z0R5	0.0001
Antithrombin III	A0N066	0.0012	Uncharacterized protein	F7FHV9	0.00011	Uncharacterized protein	F6Y7Q4	0.0001
			Putative uncharacterized protein	G7N9X1	0.00022	Uncharacterized protein	F6X9G9	0.0001
			Uncharacterized protein	F6TII3	0.00023	Uncharacterized protein	F6TII3	0.0001
			Uncharacterized protein	F6X9G9	0.00026	Prothrombin	A0N064	0.00019
			Complement C1r subcomponent	H9FZ50	0.00027	Uncharacterized protein	F7HP23	0.00024
			Uncharacterized protein	F6X837	0.00035	Gelsolin isoform a	H9Z838	0.00047
			Antithrombin III	A0N066	0.00053	Putative uncharacterized protein (Fragment)	G7N2L0	0.0007
			Putative uncharacterized protein	G7MJU4	0.0006	Alpha-1-antichymotrypsin	F6SL79	0.00088
			Fibronectin isoform 4 preproprotein	H9YY12	0.00066	Putative uncharacterized protein	G7NH09	0.0012
			Putative uncharacterized protein	G7NH09	0.00077	Putative: complement C4-A isoform 1 (Fragment)	H9FF97	0.0012
			Alpha-amylase	G7MIC0	0.00091	Uncharacterized protein	F7D6T8	0.0012
			Uncharacterized protein	F6SSW9	0.0011	Apolipoprotein(a) (Fragment)	APOA	0.0015
			Alpha-1-antitrypsin	F6SEN6	0.0012	Uncharacterized protein	F7AB32	0.0019
			Cofilin-1	I0FG05	0.0013	Apolipoprotein(a) (Fragment)	F7FPV8	0.0021
			Uncharacterized protein	F6UZ87	0.0016	Alpha-albumin (Fragment)	G7MT38	0.0024
			Thioredoxin	F6RHG3	0.0017	Putative uncharacterized protein	G7MJU4	0.0026
			Coagulation factor X protein	A0N065	0.0018	Uncharacterized protein (Fragment)	H9H2V9	0.0033
			Cytokeratin-6C	G7N720	0.0018	Annexin	F7D2H1	0.0036
			Putative uncharacterized protein	G7N2G0	0.0019	14-3-3 protein zeta/delta	F7EFF6	0.0037
			Talin-1	G7NFD2	0.0019	Amine oxidase	G7NIT9	0.0038
			Uncharacterized protein	F6YKA7	0.0019	Uncharacterized protein	F6VZJ5	0.0044
			Alpha-amylase	F7H0E4	0.002			
			Complement C3	H9EXI6	0.0025			
			Angiotensinogen preproprotein	G7MFR4	0.0027			
			Coagulation factor IX protein	A8CZ27	0.0027			
			Cytokeratin-1	F7B786	0.0031			
			Uncharacterized protein	F6TLR3	0.0034			
			Complement C3	I2CT48	0.0035			
			Annexin	F7D2H1	0.0037			
			Glutathione S-transferase P	GSTP1	0.0037			
			Tropomyosin beta chain isoform 1	F7GSX6	0.0038			
			Uncharacterized protein	F7HP23	0.0041			
			Putative uncharacterized protein (Fragment)	G7N2L0	0.0054			
			Uncharacterized protein	F6SGB4	0.0054			
			Uncharacterized protein	F7GSK8	0.0056			

Proteins were considered significant with p-values less than the Kruskal-Wallis Benjamini-Hochberg corrected values (p < 0.00179, p < 0.00622, and p < 0.00525 for days 4, 7, and 12).

Interestingly, pathway analysis revealed possible liver and intestinal damage as a consequence of gamma-irradiation (Tables 3 and 4). The LXR/RXR activation pathway is significantly activated at days 4 (4 proteins), 7 (9 proteins), and 12 (7 proteins) post-irradiation with the majority of proteins identified at day 7. LXR/RXR (liver X receptor/retinoid X receptor ligands) activation pathway has been shown previously in human intestinal cell line CaCo-2 to increase the efflux of cholesterol and plays an important role in intestinal HDL production and cholesterol absorption [6]. The liver X receptors are important regulators that control the genes involved in efflux and excretion of cholesterol and lipid homeostasis in tissues including the liver, intestine, and brain [7]. Evidence for the increased activation of these receptors indicates possible liver and intestinal damage due to gamma-irradiation. In addition, the FXR/RXR activation pathway plays a role in regulating metabolic pathways and also leads to efflux and secretion of cholesterol and was found to be activated at day 7 post-irradiation [8]. Evidence also supports the role of FXR (farnesoid X receptor) in the reduction of proinflammatory cytokines in the intestinal tract preventing inflammation and epithelial permeability [9]. The acute phase response pathway is activated for all time points and dose levels and is involved in the response to tissue injury, infection, or inflammation as well as the restoration of homeostasis [10]. Clathrin-mediated endocytosis was found to be significantly activated at day 12 post-irradiation and is involved in the clearance of LDL from the blood stream through endocytosis. Clathrin-coated vesicles form domains of the plasma membrane and are responsible for receptor-mediated endocytosis of ligands such as low density lipoprotein (LD), transferrin, growth factors, and more [11, 12]. The coagulation system and extrinsic and intrinsic prothrombin activation pathways are activated in response to vasculature damage such as bleeding and thrombosis. These pathways work in balance with one another and were identified as up-regulated at different time points and dose levels [13] (Tables 3 and 4).

**Table 3:** Time-dependent analysis.

	Dose: 6.7 Gy		Dose: 7.4 Gy	
Pathway	p-value	Overlap	p-value	Overlap
Acute Phase Response Signaling	4.98E-23	14/169	9.97E-15	10/169
Clathrin-mediated Endocytosis Signaling	9.82E-11	8/197	1.99E-07	6/197
Coagulation System	5.41E-17	8/35	4.97E-12	6/35
Extrinsic Prothrombin Activation Pathway			4.53E-09	4/16
FXR/RXR Activation	2.68E-12	8/126		
Intrinsic Prothrombin Activation Pathway			1.46E-12	6/29
LXR/RXR Activation	1.93E-12	8/121		

The top 5 canonical pathways identified by IPA from proteins significantly differentiating between days 4, 7, and 12 post-irradiation. The pathway p-values are calculated using the right-tailed Fisher Exact Test. The complete list of pathways can be found in S3 Table.

**Table 4:** Dose-dependent analysis.

	Day 4		Day 7		Day 12	
Pathway	p-value	Overlap	p-value	Overlap	p-value	Overlap
Actin Cytoskeleton Signaling			1.29E-10	9/228		
Acute Phase Response Signaling	1.83E-14	8/169	1.83E-13	10/169	8.10E-18	11/169
Clathrin-mediated Endocytosis Signaling					3.38E-13	9/197
Coagulation System	1.21E-11	5/35	1.51E-13	7/35	3.12E-10	5/35
Extrinsic Prothrombin Activation Pathway	1.03E-07	3/16			1.96E-09	4/16
FXR/RXR Activation			6.13E-13	9/126		
Intrinsic Prothrombin Activation Pathway	2.07E-09	4/29				
LXR/RXR Activation	7.17E-07	4/121	4.23E-13	9/121	3.98E-11	7/121

The top 5 canonical pathways identified by IPA from proteins significantly differentiating between 6.7 Gy and 7.4 Gy gamma-irradiation dose levels. The pathway p-values are calculated using the right-tailed Fisher Exact Test.

## Discussion

The plasma data reported here show a remarkably similar trend to what was previously reported for the analysis of urine from the same *Rhesus macaque* animals [3]. The data reported also show the importance to study the kinetics of plasma proteins change in level across doses of radiation tested (Figs 2 & 3). Furthermore, unique proteins present in the plasma are more readily detected at earlier time points for higher radiation exposures (Fig 1). Specifically, 7.4 Gy produces more unique proteins at day 4, while 6.7 Gy produces the most unique proteins at day 7. Higher radiation levels are expected to induce more severe changes which appear to translate into earlier characteristic changes to the proteome. Moreover, in the analysis of the urine from the same animals [3], the higher radiation dose (7.4 Gy) resulted in the most unique proteins on day 4 compared to the lower radiation dose (6.7Gy) in which unique proteins were at the highest level on day 7. These results differ from the radiation dose-dependent kinetics observed with citrulline for which higher radiation doses were associated with more pronounced changes at later time points [14]. The distinct kinetics of proteomic changes reported herein presents potential advantages for dosimetry applications for which an earlier assessment of exposure is critical to guide medical management. Results also suggest that the optimal time window for detection of unique proteins at each radiation dose tested is similar for the analysis of plasma and urine. A previous report using proteomics to analyze plasma from radiation accident victims identified different levels of six annotated proteins over 18 gel spots [15]; two of those six proteins were also mis-regulated in the current data set–fibrinogen alpha chain and transferrin.

**Fig 4:**
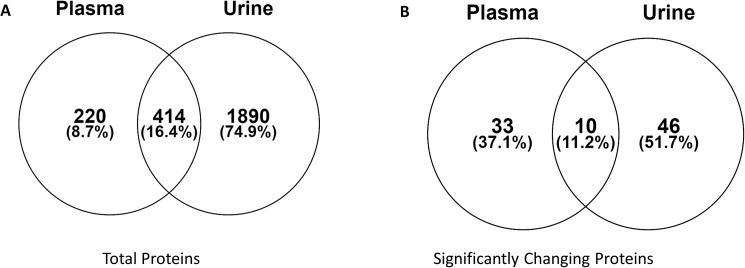
Venn diagrams show overlapping proteins identified in both plasma and urine samples from gamma-irradiated non-human primates. A) Total proteins identified in both sample types. B) From the 414 commonly identified proteins between plasma and urine, the Venn diagram shows how many were found to be significant in each sample type by ANOVA with a p-value < 0.05. Ten proteins are identified as significant from both the plasma and urine proteomic analyses.

We previously reported the identification of 2346 total proteins in urine and here we report the identification of 663 total proteins in the plasma of the same animals exposed to gamma irradiation. Of all the proteins identified in both data sets, 414 were found to be in common (Fig 4A). The Venn diagram in Fig 4B shows the number of proteins identified from both plasma and urine (414 proteins) that are also identified as significant by ANOVA (p-value < 0.05). From the list of common proteins identified in plasma and urine, 33 and 46 proteins were found to significantly change in abundance in the plasma and urine samples, respectively (Fig 4B). In addition, 10 proteins were found to significantly differentiate between both dose and time points in both plasma and urine from the same animals. These include angiotensinogen preproprotein (G7MFR4), ferritin (F6ZV45), uncharacterized proteins (F6TLR3, F7DHQ1, and F7GRY2), putative uncharacterized protein (G7NQN4 and G7MJ28), serum amyloid A (G7NDT1 and F7DY68), and angiotensin-converting enzyme isoform 1 (H9FJ99). According to UniProtKB database (www.uniprot.org), serum amyloid A is a major acute phase reactant and is the apolipoprotein of the HDL complex, uncharacterized protein F7GRY2 (C9) is involved in the immune response, and uncharacterized protein F7DHQ1 (CRP) regulates interleukin-8 secretion. Therefore, the shedding of these proteins into the plasma and urine indicate increased activity of the immune system in response to radiation exposure. The pathways involved include regulators of the efflux and secretion of cholesterol, clearance of LDL from the blood, and the coagulation system. These 10 proteins detected between the plasma and urine analyses are optimal candidates for further study as biomarkers of radiation exposure.

## Supporting information

S1 Table Normalized iBAQ intensity values. ANOVA analysis was performed in Scaffold Q+S. Proteins with a p-value < 0.05 are considered significantly different among the gamma-irradiation exposure levels and the days post-irradiation.
(XLSX)

S2 Table Raw data output from MaxQuant consisting of iBAQ, LFQ, and MS/MS count data. (XLSX)

S3 Table IPA Canonical pathways by Dose and Time-dependent analysis. The pathway p-values are calculated using a right-tailed Fisher Exact test and are shown in the -log10 scale.
(XLSX)

## References

[1] Guipaud O. Serum and Plasma Proteomics and Its Possible Use as Detector and Predictor of Radiation Diseases In: Leszczynski D, editor. Radiation Proteomics: The effects of ionizing and non-ionizing radiation on cells and tissues. Dordrecht: Springer Netherlands; 2013 p. 61–86. 10.1007/978-94-007-5896-4_4 23378003

[2] Schley G, Köberle C, Manuilova E, Rutz S, Forster C, Weyand M, et al Comparison of Plasma and Urine Biomarker Performance in Acute Kidney Injury. PLoS ONE. 2015;10(12):e0145042 10.1371/journal.pone.0145042 26669323 PMC4682932

[3] Byrum SD, Burdine MS, Orr L, Moreland L, Mackintosh SG, Authier S, et al A Quantitative Proteomic Analysis of Urine from Gamma-Irradiated Non-Human Primates. Journal of proteomics & bioinformatics. 2016;9(Suppl 10):005. 26962295 10.4172/jpb.S10-005 PMC4780756

[4] Schwanhausser B, Busse D, Li N, Dittmar G, Schuchhardt J, Wolf J, et al Global quantification of mammalian gene expression control. Nature. 2011;473(7347):337–42. 10.1038/nature10098 21593866

[5] Krey JF, Wilmarth PA, Shin J-B, Klimek J, Sherman NE, Jeffery ED, et al Accurate Label-Free Protein Quantitation with High- and Low-Resolution Mass Spectrometers. Journal of Proteome Research. 2014;13(2):1034–44. 10.1021/pr401017h 24295401 PMC3946283

[6] Murthy S, Born E, Mathur SN, Field FJ. LXR/RXR activation enhances basolateral efflux of cholesterol in CaCo-2 cells. Journal of Lipid Research. 2002;43(7):1054–64. 12091489 10.1194/jlr.m100358-jlr200

[7] Hong C, Tontonoz P. Liver X receptors in lipid metabolism: opportunities for drug discovery. Nat Rev Drug Discov. 2014;13(6):433–44. 10.1038/nrd4280 24833295

[8] Ding L, Pang S, Sun Y, Tian Y, Yu L, Dang N. Coordinated Actions of FXR and LXR in Metabolism: From Pathogenesis to Pharmacological Targets for Type 2 Diabetes. International Journal of Endocrinology. 2014;2014:13. 10.1155/2014/751859 PMC4020365 24872814

[9] Stojancevic M, Stankov K, Mikov M. The impact of farnesoid X receptor activation on intestinal permeability in inflammatory bowel disease. Canadian Journal of Gastroenterology. 2012;26(9):631–7. 22993736 10.1155/2012/538452 PMC3441172

[10] Moshage H. Cytokines and the hepatic acute phase response. The Journal of Pathology. 1997;181(3):257–66. 10.1002/(SICI)1096-9896(199703)181:3<257::AID-PATH756>3.0.CO;2-U 9155709

[11] Marsh M, McMahon HT. The Structural Era of Endocytosis. Science. 1999;285(5425):215–20. 10398591 10.1126/science.285.5425.215

[12] Anderson RGW, Brown MS, Goldstein JL. Role of the coated endocytic vesicle in the uptake of receptor-bound low density lipoprotein in human fibroblasts. Cell. 1977;10(3):351–64. 191195 10.1016/0092-8674(77)90022-8

[13] Palta S, Saroa R, Palta A. Overview of the coagulation system. Indian Journal of Anaesthesia. 2014;58(5):515–23. 10.4103/0019-5049.144643 25535411 PMC4260295

[14] Bujold K, Hauer-Jensen M, Donini O, Rumage A, Hartman D, Hendrickson HP, et al Citrulline as a Biomarker for Gastrointestinal-Acute Radiation Syndrome: Species Differences and Experimental Condition Effects. Radiation Research. 2016;186(1):71–8. 10.1667/RR14305.1 27351760 PMC4976929

[15] Nylund R, Lemola E, Hartwig S, Lehr S, Acheva A, Jahns J, et al Profiling of low molecular weight proteins in plasma from locally irradiated individuals. Journal of Radiation Research. 2014;55(4):674–82. 10.1093/jrr/rru007 24570173 PMC4099999

